# Insights into drug resistance in *Leishmania*: Mechanisms, therapeutics, and clinical case studies

**DOI:** 10.5599/admet.2992

**Published:** 2026-01-14

**Authors:** Gajala Deethamvali Ghousepeer, Mansi Rani, Aman Kumar, Shubhankar Kumar Singh, Anjali Priyadarshini, Elcio Leal, Shailja Singh, V. Samuel Raj, Subhajit Basu, Ramendra Pati Pandey

**Affiliations:** 1 School of Health Science and Technology, UPES, Dehradun, India; 2 Indian Council of Medical Research-Rajendra Memorial Research Institute of Medical Science, Patna, India; 3 Centre for Drug Design Discovery and Development (C4D), SRM University, Delhi-NCR, India; 4 Special Center for Molecular Medicine, Jawaharlal Nehru University, Delhi, India; 5 Laboratório de Diversidade Viral, Instituto de Ciências Biológicas, Universidade Federal do Pará, Belem, 66075-000, Brazil; 6 School of Biosciences and Bioengineering, D Y Patil International University, Pune, India

**Keywords:** Leishmaniasis, neglected tropical disease, immune responses, treatment

## Abstract

**Background and purpose:**

Leishmaniasis, a neglected tropical disease caused by the protozoan parasite *Leishmania*, remains a significant public health concern in endemic regions. The disease manifests in various forms, including cutaneous, mucocutaneous, and visceral leishmaniasis, each associated with specific *Leishmania* species and influenced by host immune responses. Over the past few decades, treatment for leishmaniasis has relied on a limited range of drugs, including pentavalent antimonials, amphotericin B formulations, miltefosine, and paromomycin. However, widespread drug resistance, particularly in visceral leishmaniasis, has severely compromised treatment efficacy, leading to rising cases of treatment failure. This review aims to provide a comprehensive understanding of the mechanisms underlying drug resistance in leishmaniasis and to highlight the factors that contribute to its development.

**Experimental approach:**

The study synthesizes existing literature on resistance mechanisms among anti-leishmanial drugs, focusing on changes in parasite uptake and efflux, intracellular sequestration, and modulation of stress responses. It also examines the impact of environmental factors, such as arsenic exposure in endemic regions, and reviews recent molecular and genomic studies that have identified resistance-associated markers.

**Conclusion:**

The review underscores the urgent need for innovative therapeutic strategies and highlights the importance of an integrated approach to combat drug resistance through enhanced surveillance, molecular insights, and global collaboration.

## Introduction

Leishmaniasis, a zoonotic vector-borne disease, is caused by a flagellated protozoan parasite of the genus *Leishmania*. This dimorphic intracellular parasite is transmitted by the bite of a *phlebotomine* (Old World) sandfly vector to its mammalian host, with over 90 species capable of transmitting the disease. In the New World, the primary vector, *Lutzomyia spp*., transmits infection. More than 20 species of the *Leishmania* protozoan parasite are expected to cause infectious disease. These parasites can inhabit two different hosts, each with distinct morphological changes. Clinical features vary with the *Leishmania* species involved and the host's immune response. Including tropical, sub-tropical, and in a few European countries, these parasitic diseases are found in more than 98 countries, and most developing countries are at risk [1]. Each year, more than 1 million cases of these diseases are reported. Mostly, the poorest people and people in rural areas with a lack of sanitation, poor housing, and weak immune systems are prone to this disease [2].

Globally, different countries from Asia, Africa, and America are greatly affected by this endemic parasitic disease. The clinical description of this endemic disease was first observed in Aleppo, Syria, by Alexander Russell in 1756 and was called the “Aleppo Boil”. In 1885, Sergeant Major Cunningham of the Indian Medical Service in Calicut reported a parasitic organism collected from tissue samples of a patient with a “Delhi boil” [3]. Leishmaniasis has three different clinical manifestations: cutaneous leishmaniasis (CL), mucocutaneous leishmaniasis (ML), and visceral leishmaniasis (VL). Among these three clinical forms of leishmaniasis, visceral leishmaniasis is considered the most fatal if left untreated. In the Indian Subcontinent, it is called “Kala Azar” (which means black fever). The VL primarily affects the spleen, liver, bone marrow, and lymph nodes, which serve as reservoirs of lymphocytes that facilitate the parasites' survival during the disease. A total of 90,312 new cases of visceral leishmaniasis (VL) were recorded globally as of 2021, according to the World Health Organization (WHO) Weekly Epidemiological Record [4,5]. *Leishmania donovani* and *Leishmania infantum* are the two main species that cause VL. cutaneous leishmaniasis is also one of the prevalent clinical disease forms, which is characterized by ulcerative skin lesions and multiple non-ulcerative nodules, primarily developing at the site of *Phlebotomus* sand fly bites [6], with 70 countries that are endemic with CL, 90 % of the disease has spread across Asian countries [7]. In mucocutaneous leishmaniasis, both the skin and mucous membranes are infected [8], and lesions can be visible on the upper lip and nose during the mucocutaneous phase [9]. Depending on the host's immune system, clinical manifestations and symptoms vary with the *Leishmania* species involved [10].

**Figure 1.**
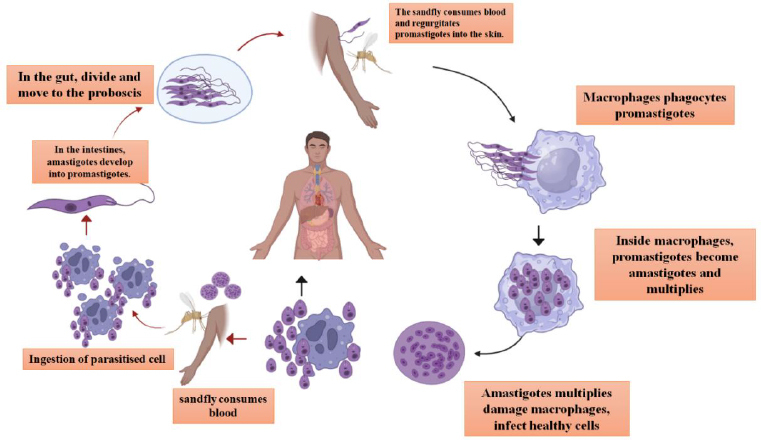
The life cycle of *Leishmania donovani*

As leishmaniasis is considered a neglected tropical disease, it has a digenetic life cycle, which enables this species to adapt to both host mammalian cells and insect vectors [11]. If the host is an animal, the infection is classified as zoonotic leishmaniasis, which sustains the parasite transmission cycle among animals such as goats, dogs, and rodents [12]. If the host is human, then it is called anthroponotic leishmaniasis [1]. Two distinct morphological forms of *Leishmania* have been reported: parasitic promastigotes, an extracellular form in the insect vector, and the intracellular amastigote form in host mammalian cells. Initially, upon taking a blood meal from the host, the sandfly injects its saliva into the blood to prevent clotting. Subsequently, the sand fly vector transmits the metacyclic promastigote form into the host by penetrating mammalian skin and invading neutrophils. Along with the neutrophils, metacyclic promastigotes also infect Langerhans cells and fibroblasts [13]. At the site of infection, the macrophages invade and engulf the promastigote, where these parasites attach to the macrophages and get internalized into a parasitophorous vacuole, which is phagocytosed by the macrophages [14]; within the macrophage, the amplification, along with the differentiation of the promastigote form into amastigote form, takes place [15]. While some amastigotes attach to the vacuolar membrane, others remain free within the vacuole and undergo several rounds of asexual multiplication [16]. The parasites are released into surrounding tissues, primarily via macrophage death mechanisms, including apoptosis and necrosis, rather than via active host membrane bursting [17]. These amastigotes can either infect fresh macrophages or be consumed by a fresh female phlebotomine when it feeds on blood [15], as shown in Figure 1.

**Figure 2.**
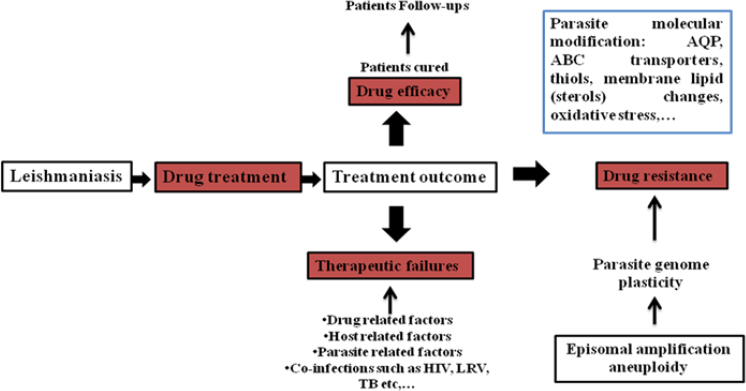
Factors leading to treatment failure and drug resistance in leishmania [20] (HIV: Human immunodeficiency virus, LRV: *Leishmania* RNA virus, TB: Tuberculosis, AQP: Aquaporin)

The current therapeutic approach to leishmaniasis involves the administration of several recommended drugs, including sodium stibogluconate, pentamidine, amphotericin B, Liposomal amphotericin B, miltefosine, and paromomycin. Many alternatives, such as plant-derived compounds, have also been studied and reported as potential sources of new anti-leishmanial therapeutics [18]. Over the past 25 years, the therapeutic efficacy of several anti-leishmanial drugs has been significantly compromised due to the emergence and widespread establishment of resistance among *Leishmania* strains [19]. This has led to an increase in treatment failure among patients across the endemic regions, with emerging cross-resistance. Although treatment failure and drug resistance are not directly linked, multiple factors influence treatment outcomes, including the host immune system, the parasite, and the environment, as shown in Figure 2 [20]. Treatment failure due to the increasing drug resistance poses a major challenge to current treatment regimens, which shifts the focus to developing alternative therapeutic strategies. This review aims to assess the drug resistance that has been reported against anti-leishmanial drugs and the studies on drug resistance mechanisms and this is discussed along with case studies reporting treatment failures in patients.

## Therapeutics, strategies and challenges

Preventing mortality and morbidity are the main objectives of treatment for all types of leishmaniasis. Few approved drugs are listed in Table 1; however, there is no immunoprotection and no current vaccine therapies or chemoprophylaxis available against leishmaniasis. Several compounds and formulations exhibit anti-leishmanial properties. For primary treatment, pentavalent antimonials are the first-line drugs used to treat all forms of leishmaniasis. These drugs have not been approved by the FDA 10 [21]. The only leishmaniasis treatments approved by the FDA are oral miltefosine for CL, ML, and VL caused by certain species, and intravenous liposomal amphotericin B (L-AmB) for VL [22].

**Table 1.** List of approved medications for leishmaniasis [23]

Drug name	Generic name	Drug class	Brand names	Treatment
pentavalent antimony	sodium stibogluconate (SSG)	pentavalent antimonials	Pentostam®	visceral, cutaneous, and mucosal forms of *Leishmaniasis* and PKDL
amphotericin b	amphotericin B systemic	polyenes	Amphocin, Fungizone	visceral leishmaniasis or Kala-azar, second-line treatment for mucosal leishmaniasis and cutaneous leishmaniasis
amphotericin B liposomal	amphotericin B liposomal systemic	polyenes	AmBisome	visceral leishmaniasis or Kala-azar, second-line treatment for mucosal leishmaniasis and cutaneous leishmaniasis
Miltefosine	miltefosine systemic	anthelmintics	Impavido	visceral, cutaneous, and mucosal forms of leishmaniasis
pentamidine, pentamidine isethionate, pentamidine mesylate	pentamidine systemic	miscellaneous antibiotics, inhaled anti-infectives	Pentam, Nebupent, Pentam 300, Pentacarinat®, Lomidine®	visceral, cutaneous, and mucosal forms of leishmaniasis
amphotericin B lipid complex	amphotericin B lipid complex systemic	polyenes	Abelcet	visceral leishmaniasis or Kala-azar, second-line treatment for mucosal leishmaniasis and cutaneous leishmaniasis
paromomycin sulfate, paromomycin sulfate (15 %) ointment	humatin	aminoglycoside	Leshcutan®	visceral, cutaneous forms of leishmaniasis

Sodium stibogluconate (SSG) and meglumine antimoniate, both pentavalent antimonials approved by the WHO, are first-line treatments for leishmaniasis. Pentavalent antimonials are effective in a few parts of the world, particularly in Africa, and are restricted due to their high toxicity, particularly cardiotoxicity. During stibogluconate therapy, HIV-positive individuals experience more severe side effects, decreased efficacy, and increased mortality [24,25]. From the beginning of treatment with pentavalent antimonials in India until the 80s, treatment failure due to pentavalent antimonial resistance was observed. To overcome these problems, pentamidine was used to treat antimony-resistant VL [26]. However, its use is restricted because of well-known toxicities, which include hypotension, heart problems, gastrointestinal adverse effects, diabetes mellitus, and pancreatitis that results in insulin-dependent diabetes [27]. Due to these drawbacks, the other drug, the polyene antibiotic amphotericin B deoxycholate, was widely used for VL and is also advised for the treatment of PKDL in the Indian subcontinent, which is resistant to antimonial therapy and is recommended over pentamidine [28]. Even if the therapy works effectively, the side effects, such as nephrotoxicity, myocarditis, and hypokalemia, make it necessary to monitor the patient [26].

To overcome these adverse effects, lipid formulations of the drugs, which substitute alternative lipids for deoxycholate, such as liposomal amphotericin B, amphotericin B lipid complex, and amphotericin B cholesterol dispersion, were developed. These formulations are rapidly absorbed by the liver, spleen, and other organs, and are concentrated in the reticuloendothelial tissues, where the disease occurs in patients with VL. It persists there for a long time, allowing high doses of the medication to be administered quickly. Nephrotoxicity is reduced since it spares vital organs such as the kidneys. Several lipid formulations of amphotericin B have been thoroughly investigated for leishmaniasis; of them, only liposomal amphotericin B (L-AmB) has received US FDA approval. Liposomal amphotericin B dosages can vary by region and area [29].

An alkyl phospholipid compound was discovered and developed as an oral medication for the treatment of solid tumours in the early 1980s by two scientific groups, one from Germany investigating anti-tumour activity and the other from the UK investigating anti-inflammatory properties [30,31]. However, the drug was discontinued due to dose-limiting gastrointestinal adverse events in multiple clinical studies. Later, this compound, known as miltefosine, was repurposed as an antileishmanial agent. This first oral drug has shown excellent oral absorption in mouse models and superior efficacy compared with intravenous pentavalent antimonials in animal studies [32,33], suggesting that miltefosine is a promising candidate for the treatment of human VL [34].

Paromomycin previously existed as a paromomycin sulfate [35] and is an aminoglycoside antibiotic that originated from the bacterial pathogen *Streptomyces rimosus Paromomycin w*as also considered for the treatment of parasitic infestation. It has been established that treating patients with visceral leishmaniasis is safe and effective. This medication is cheap and approved as a first-line substitute when traditional anti-leishmanial medications are no longer effective.

## Drug resistance in *Leishmania*

Multiple cases among the patients, along with the experimental studies, have demonstrated that *Leishmania* parasites can develop resistance to all available drugs. Initial drug resistance was reported in the sodium stibogluconate, which was the first line of the drug to treat leishmaniasis, and due to this resistance, it has led to treatment failure in patients from endemic regions [36]. As an alternative to sodium stibogluconate, an oral drug, miltefosine has been used; however, in the past few decades, miltefosine has shown limited efficacy in South Asian countries such as India [37] and Nepal [38], which were subsequently replaced by liposomal amphotericin B. For clinical resistance, the problem of resistance in these parasites can arise experimentally for any drug. Under stressful conditions, parasites undergo genetic mutations, which ultimately help them overcome drug resistance. This is a basic representation of the resistance mechanism, but it may not occur in all parasites. Anti-leishmanial treatments vary across regions, and drugs are recommended accordingly. Evidence shows that *Leishmania* extracellular vesicles mediate horizontal gene transfer, aiding the transmission of drug-resistance genes and enhancing parasite fitness under stress or during drug exposure in hostile environments [39].

### Pentavalent antimonials

**Figure 3.**
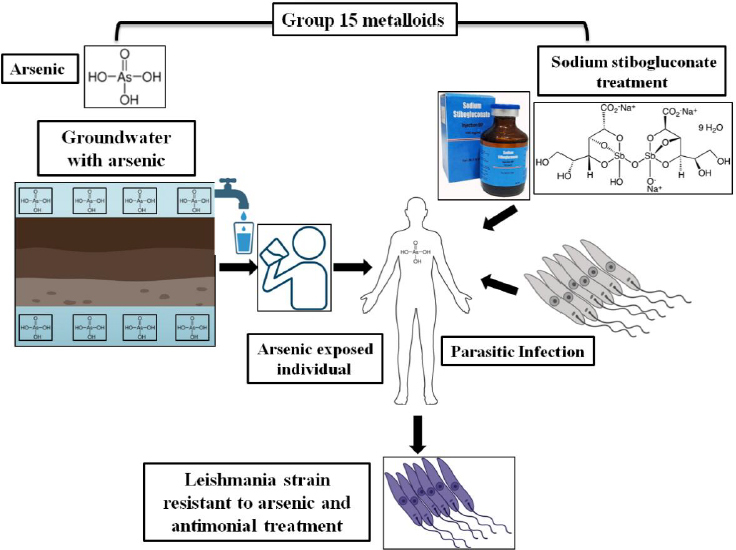
Schematic figure representing the parasites exposed to antimonials along with groundwater arsenic in Bihar

In the late 1970s, India experienced a significant increase in sandfly populations, resulting in a visceral leishmaniasis epidemic. Despite increasing dosages and extended treatment durations, the efficacy of antimonial therapies steadily declined. Few cases of parasites resistant to sodium stibogluconate have been reported [40]. A study revealed antimony and arsenic which are Group 15 metalloids, exhibit similar structural and chemical properties [41], whereas in the Indian subcontinent high concentrations of arsenic naturally occurring in groundwater have been accumulated by tube wells which have been found in endemic region Bihar [42], where it is believed that 90 % of India's VL cases have been reported [43]. If a person from this endemic region who is chronically exposed to arsenic contracts VL, these parasites are also readily exposed to arsenic, as they reside within the lymphoreticular organs [44]. This might lead to the emergence of a Leishmania strain resistant to arsenic, rendering it resistant to antimonial treatment, as shown in Figure 3. This study in Bihar found a 59 % SSG failure rate, with higher failure rates associated with arsenic-contaminated groundwater [45].

SSG, a pentavalent antimonial compound, is an antileishmanial compound. Instead of being a single molecule, SSG is a chemically produced combination developed by the reaction of gluconic and stibonic acids [46]. The parasite resists antimonials by modulating its cell influx. Sb(V) is reduced to Sb(III), partially in the host macrophage, and enters via the AQP1 carrier. An unknown mechanism facilitates Sb(V) entry into the parasite, where it further reduces Sb(III), and Sb(V) and Sb (III) accumulate in the promastigote and amastigote forms of *Leishmania*; however, their entry routes differ. Accumulation levels don't correlate with antimony susceptibility, suggesting other factors in drug action. Resistant parasites accumulate less antimony than sensitive ones, but the correlation between accumulation levels and sensitivity in wild-type cells is unclear [47]. Down-regulation of AQP1 is also linked to drug resistance in Indian VL and PKDL isolates, correlating with Sb(III) accumulation, with some exceptions [48]. When AQP1 is overexpressed, the parasites become extremely sensitive to Sb(III) [49]. In India, treatment for visceral leishmaniasis (VL) is hampered by antimony (Sb) insensitivity. *In vitro* investigations reveal elevated thiols and drug efflux pumps in Sb-resistant parasites. Through reduced oxidative stress, this study identified clinical isolates with higher thiol levels and faster regeneration, which contribute to Sb resistance [50]. Ultimately, this antimony causes oxidative stress in cells, and resistance arises from the parasite’s ability to manage this stress.

Antimony-resistant parasites show increased intracellular trypanothione levels and a higher thiol redox potential, linked to the overexpression of key enzymes in glutathione and polyamine synthesis, namely gamma-glutamylcysteine synthetase and ornithine decarboxylase [51]. When it comes to the *Leishmania* ATP-binding cassette P-glycoprotein A (PGPA), which confers arsenite and antimonite resistance by sequestering metal-thiol conjugates, its exact antimony resistance mechanism is unclear [52].

**Figure 4.**
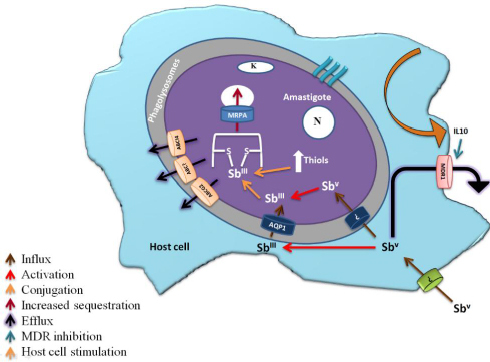
Molecular mechanism of Drug Resistance against pentavalent antimonials (The figure shows an amastigote within a macrophage's phagolysosome, detailing how antimonials enter the parasite and the intracellular processes that lead to drug resistance. It highlights the roles of ABC transporters, aquaporins (AQP), and proteins involved in drug resistance (DR), including MDR1 (Multidrug Resistance Protein 1) and interleukin 10 (IL-10) [20].

Drug resistance is conferred by another resistant protein of the ABC transporter MRPA (ABCC3/PgpA), which sequesters drug-trypanothione conjugates in an organelle close to the flagellar pocket. At this site, antimonial targets are expected to be absent [52]. At the flagellar pocket, exocytosis is used to eject these conjugates. Furthermore, TSH-antimonial substances are actively effluxed by a surface protein [53]. Overexpression of ABC transporters in *Leishmania* influences drug efflux and antimony resistance. MRPA, an ABC transporter, is localized in membrane vesicles near the flagellar pocket [54]. The diagrammatic explanation of the molecular mechanism of antimonial drug resistance is shown in Figure 4.

Pentamidine resistance protein 1(PRP1), which is a P-glucoprotein/ ATP-binding cassette transporter protein, can provide resistance against pentamidine when overexpressed in *Leishmania major* [55]. ABCI4 is also an intracellular ATP-binding cassette transporter in *L. major*, involved in heavy metal export and resistance to antimonials. It reduces mitochondrial toxicity and reactive oxygen species, enhancing parasite replication and drug resistance [56]. Another ATP-binding cassette transporter, LABCG2, contributes to antimony resistance by reducing Sb(III) accumulation and facilitating drug efflux [57]. In another study, antimony-resistant *Leishmania donovani* (SbRLD) increases IL-10 and MDR1 (multidrug resistance protein 1) levels in infected macrophages. SbRLD's unique glycan, which can be removed, influences this process. SbRLD engages Toll-like receptors to activate pathways that upregulate IL-10 and MDR1, with IL-10 being crucial for MDR1 induction [58]. Antimony resistance in *Leishmania* arises from various factors and events, complicating treatment outcomes. Understanding these mechanisms, observed in labs, helps explain the rise and spread of resistance to SSG.

### Amphotericin-B

**Figure 5.**
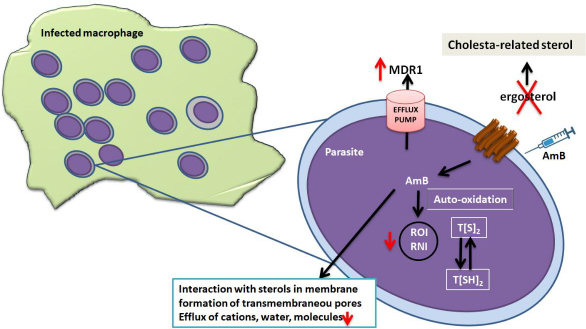
The mechanism of action of amphotericin and *Leishmania* resistance mechanisms are shown in red. (AmB, Amphotericin B; RNI, reactive nitrogen intermediates; ROI, reactive oxygen intermediates; T[SH2], trypanothione; TR, trypanothione reductase.

A polyene antibiotic, amphotericin B deoxycholate, was widely used for VL and is also recommended for the treatment of PKDL in the Indian subcontinent, in preference to antimonial therapy due to resistance, and is recommended over pentamidine [28]. As reported, amphotericin B (AmB) binds to ergosterol and episterol in the parasite's membrane [59]. The amphipathic structure of AmB enables the hydrophobic portion to interact with membrane lipids, whereas the hydrophilic portion forms pores that increase membrane permeability and lead to cell death. In fact, they substitute ergosterol for cholesterol-associated sterols, decreasing the binding affinity of AmB [60-62]. These changes also increase membrane fluidity and AmB efflux, due to upregulation of the MDR1 efflux pump, making the parasites more tolerant to oxidative stress. The effectiveness of amphotericin B for treating visceral leishmaniasis in Bihar, India, is at risk due to emerging drug resistance. A resistant *Leishmania donovani* strain requires an 8-fold higher dose to be lethal compared to a sensitive strain. This resistance is linked to elevated thiol levels and enhanced thiol metabolic pathways. These factors collectively contribute to resistance, which can be partially reversed using inhibitors targeting thiol metabolism and ABC transporters, as shown in Figure 5 [60].

### Miltefosine

**Figure 6.**
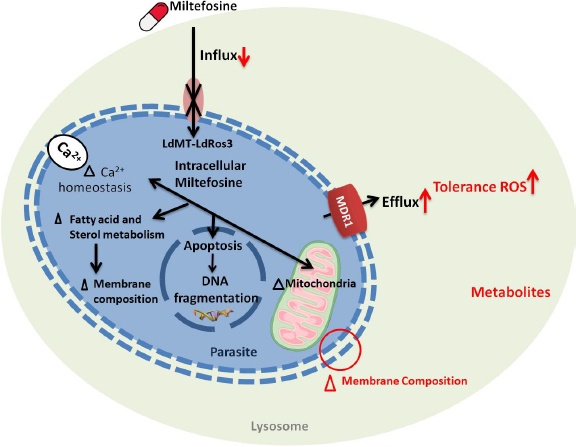
The *Leishmania* modes of action of miltefosine, along with the potential mechanism of resistance against miltefosine. (MDR, multidrug resistance transporter; LdROS3, a subunit of the LdMT transporter; *Leishmania donovani* miltefosine transporter).

Miltefosine, an alkyl phospholipid with antileishmanial properties, has been shown to cause treatment failure in *L. pneumonia* infections [63]. Multiple relapsed cases of VL have been reported in the Indian subcontinent when miltefosine was used as a mono-therapy [37]. Clinical isolates of *Leishmania donovani*, isolated from both cured and relapsed patients, showed similar *in vitro* susceptibility [64], indicating that treatment failure is not directly related to the parasite's intrinsic sensitivity to miltefosine. Treatment failure in these clinical isolates has been associated with increased tolerance to anti-leishmanial drugs such as amphotericin B (AmB), due to the absence of ergosterol (increased membrane fluidity and reduced AmB binding), reduced infectivity, and resistance to oxidative stress. AmB-resistant strains showed enhanced efflux by up-regulating the MDR1 (multidrug resistance) efflux pump. Miltefosine also disrupts intracellular Ca^2+^ homeostasis, a significant target of drug action in trypanosomatids [65]. Other studies have revealed that developing miltefosine resistance in the isolates of *Leishmania in vitro* led to a mutation in two genes, ROS3 and MT, which encode the *Leishmania* miltefosine transporter complex (LdMT) [66], as this complex helps in the translocation of phospholipid across the parasite's membrane and is responsible for miltefosine uptake (Figure 6) [67].

### Paromomycin

**Figure 7.**
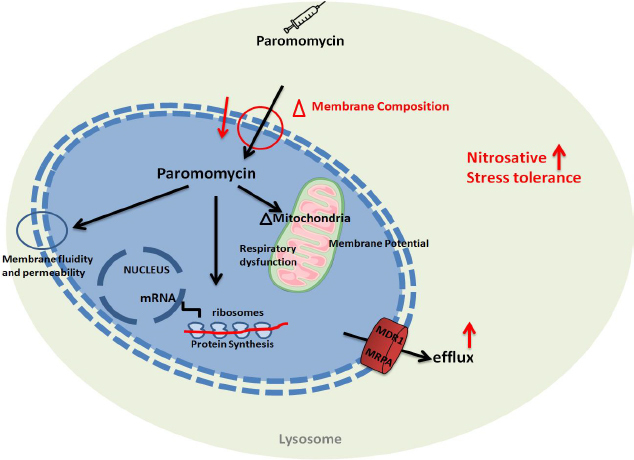
The mechanism of action of paromomycin involves inhibiting protein synthesis in *Leishmania* by binding to the ribosomal RNA. Possible resistance mechanisms in *Leishmania* include overexpression of the multidrug resistance transporter 1 (MDR) and multidrug resistance-associated protein A (MRP1), which may reduce drug efficacy by decreasing intracellular drug concentration.

Paromomycin, a cost-effective aminoglycoside antibiotic, is considered among the safest antileishmanial drugs. It generally causes only mild adverse effects, including injection site pain and cytotoxicity, making it a favourable treatment option for leishmaniasis [68]. The mechanisms of action and resistance to paromomycin have been studied in bacteria, but in the parasite, they remain poorly understood. Many studies reported that paromomycin impairs mitochondrial function in *Leishmania donovani* promastigotes, leading to a decline in energy supply due to a disturbance in the proton gradient generated by respiration [69]. Regarding the resistance mechanism, paromomycin (PMM) resistance in *Leishmania donovani* is associated with altered membrane fluidity, decreased intracellular drug accumulation, increased expression of ABC transporters, and greater tolerance to host defense mechanisms, such as nitrosative stress and complement-mediated lysis. Despite these changes, the PMM-resistant parasites remain susceptible to other antileishmanial agents, such as sodium antimony gluconate and miltefosine [70]. In other resistance mechanism studies [71], the paromomycin is associated with the parasite’s cell surface, binding to the glycocalyx, and ultimately disrupting mitochondrial function. The drug inhibits protein synthesis in both cytoplasmic and mitochondrial compartments. Additionally, resistance to paromomycin is associated with reduced drug accumulation and less pronounced effects on membrane potential and protein synthesis, suggesting that resistance involves alterations in drug binding and uptake mechanisms. The paromomycin cure rate among patients has decreased over time. In Southeast Asia, a phase IV trial reported cure rates of over 94 % with a systemic dose of 11 mg/kg for 21 days against *L. Donovani* [72,73]. However, in 2010, the same dose was insufficient in East Africa, where higher doses of 20 mg/kg or more, along with prolonged treatment periods of 28 days, were required to achieve cure rates of up to 84.3 % [74,75]. Please refer to Figure 7.

## New therapeutic approaches

Multiple new therapeutic approaches are being developed for the effective treatment of leishmaniasis. To understand the drug-resistance mechanism and to support drug discovery, researchers developed a genome-wide CRISPR-Cas9 screening system for *Leishmania infantum* [76]. These researchers identified genes associated with resistance to miltefosine and amphotericin B, notably the miltefosine transporter, sterol 24C methyltransferase, and other membrane or hypothetical proteins. Disrupting these genes confirmed their role in drug resistance. The study demonstrates that genome-wide CRISPR-Cas9 screens are feasible in *Leishmania* and can greatly accelerate the search for new antileishmanial drug targets. Most of the patients across Asian countries with leishmaniasis as an endemic experience disease relapse within 6-12 months after treatment. To overcome relapse, many researchers are developing multidrug therapies, which are expected to become more common in leishmaniasis, as they can shorten treatment duration, reduce costs and toxicity, lower hospitalization needs, improve patient compliance, and minimize the risk of drug resistance [77]. Multiple promising vaccine candidates are also under scrutiny against visceral leishmaniasis. In a recent study, the LACK protein in leishmaniasis induced a Th2-biased, nonprotective immune response in BALB/c mice, underscoring its ineffectiveness as a standalone visceral leishmaniasis vaccine [78]. In another vaccination study, a novel multiple epitope vaccine for *Leishmania* incorporating a Wolbachia surface protein (WSP) derived from *Wolbachia* bacteria has been developed, which is an adjuvant effectively engaged TLR2/TLR4 and induces a strong Th1 immune response with elevated IFN-γ, IL-12, and IL-2, and generated memory T cells, demonstrating promising immunogenicity for visceral leishmaniasis prevention [79]. Another vaccine candidate, LinKAP, a novel mitochondrial-associated protein identified from Leishmania associated with Poly ICLC, significantly reduced parasite burden in mice and hamsters, demonstrating potent prophylactic and therapeutic efficacy against visceral leishmaniasis and highlighting its promise as a vaccine candidate [80].

## Reported case study

Multiple case studies have been reported worldwide in which patients have not responded to anti-leishmanial treatment. There could be multiple reasons for such treatment failures among patients, including host immunological factors, parasite resistance, dose-dependent resistance within the species, route of drug administration, Inappropriate initial treatment, and, in some cases, mixed infection with two strains [81]. Multiple cases are presented in Table 2.

**Table 2.** Treatment failure case reports among the 5 continents: CL- cutaneous leishmaniasis; VL- visceral leishmaniasis; MCL- mucocutaneous leishmaniasis; DCL- diffuse cutaneous leishmaniasis; FLU- fluconazole; GLU- meglumine antimoniate (Glucantime®); sodium stibogluconate (Pentostan®); PENT- pentamidine; PAR- paromomycin; AmB- amphotericin B; L-AmB- liposomal amphotericin B; MIL- miltefosine; IL- intralesional; IM- intramuscular

Region	Patients	Clinical manifestation	Species	Unsuccessful treatment	Proposed mechanisms for failure/relapse	Ref.
Asia	Male (6)	CL(3), VL(3)	*L. tropica* (CL), *L. donovani* (VL)	SS, AmB, AmB-L, MIL, GLU IL and GLU IM	CL- hosts immunological factors and parasite resistance, VL- hosts immunological factors and parasite resistance	[82-86]
South America	Male (21), Female (2)	CL(15), VL(6), MCL(1), DCL(1)	*L. guyanensis* (CL), *L. panamensis* (CL), *L. naiffi* (CL), *L. tropica* (CL), *L*. *braziliensis* (CL), *L*. *infantum* (VL), *L. braziliensis* (MCL), *L. amazonensis* (DCL)	SS, AmB, AmB-L, MIL, GLU IL and GLU IM, AmB e PENT / GLU e AmB, PENT (IL or IM)	CL- dose-dependent resistance of the species, route of drug administration, Inappropriate initial treatment, presence of LRV virus, VL- not reported, MCL-host immunelogical factors, DCL- host immunological factors	[87-96]
North America	Male (1)	VL(1)	NR	GLU	VL- inappropriate initial treatment	[97-103]
Europe	Male (4) Female (6)	CL(2), VL(8)	*L. infantum* (VL), *L. tropica* (CL),	L-AmB e AmB lipid complex, MIL e AmB, GLU	CL- inappropriate initial treatment, VL- host immunological factors, mixed infection by two strains	[94]
Africa	Male (1) Female (1)	VL(2)	*L. infantum* (VL)	GLU, AmB IV, GLU / L-AmB, L-AmB, L-AmB+MIL / L-AmB, L-AmB+MIL / L-AmB and GLU / L-AmB	VL- host immunological factors	[103]

### Asia

When it comes from Asian countries that have declared leishmaniasis as endemic in those regions, where multiple treatment failures have been reported. A case study discusses a 31-year delayed recurrence of leishmaniasis, likely triggered by local trauma and topical steroids. In cutaneous leishmaniasis caused by *Leishmania tropica*, the parasite appears to persist at the original infection site, reactivating in response to changes in the local immune environment. While TGF-β may play a role in reactivation, IL-10 was not detected. The patient responded well to treatment with itraconazole and local sodium stibogluconate, and healing was achieved after stopping steroids. The case suggests that leishmaniasis might never be fully cured but is instead controlled by a balanced immune response involving both CD4 and CD8 T cells [82]. India, where it contributes 20 % of the global VL cases, has shown treatment failure with single-drug therapies, and due to the co-infection with HIV, it has become difficult to treat the patients. In a case study reported by S. Patole *et al.* [83], a patient who had been infected by VL and had a co-infection of HIV was initially treated with mono therapies of miltefosine but eventually, the disease relapse has been showing multiple times, even after different regimens of liposomal amphotericin B. due to the immune suppression and the synergistic effects of HIV and VL single-drug therapies often fail.

In a case study [84], an 8-year-old Syrian boy experienced lip swelling and lesions over four years when he was diagnosed with *Leishmaniasis Recidiva Cutis* (LRC). Initially, he was treated with intralesional meglumine antimoniate, but the treatment was ineffective; scarring persisted, and new papules subsequently developed. Later, the lesions were improved significantly after a month of treatment with systemic meglumine antimoniate, oral fluconazole, and cryotherapy. Similarly, another case study [85] describes a Palestinian Bedouin child who was diagnosed with *Leishmaniasis recidivans*, a rare form of cutaneous leishmaniasis characterized by chronic, recurring skin lesions. The child underwent multiple treatment rounds with sodium stibogluconate, but relapses occurred, possibly due to therapy non-compliance or drug-resistant strains. Traditional treatments were considered but not pursued. This emphasizes the importance of diagnostic precision and tailored treatments for cases of resistant or recurring leishmaniasis.

A case study [86] where visceral leishmaniasis (VL) relapses in a patient after miltefosine treatment is reported. The patient initially responded well to the 28-day miltefosine course, with no detectable *Leishmania* at discharge. However, ten months later, symptoms returned, including high fever and splenomegaly. Diagnostic tests confirmed VL relapse, prompting treatment with amphotericin B, which led to complete recovery. This case highlights the potential for VL relapse even after full miltefosine treatment. In another case study [88], an 80-year-old immunosuppressed man was diagnosed with visceral leishmaniasis (VL) 15 years after exposure. Despite receiving standard treatment with liposomal amphotericin B, his condition deteriorated, ultimately leading to death. The case emphasizes the unusually long latency of VL in a non-endemic area, diagnostic challenges due to atypical presentation, and treatment failure with amphotericin B. The report suggests considering pentavalent antimony as an alternative treatment for cases resistant to amphotericin B.

### North and South America

Brazil accounts for more than 99% of the estimated 3,500 annual cases in Latin America. While the number of cases remains stable, the spread is extending southwest across the continent [5]. Multiple case reports have been reported in South America, including one [87] detailing the failure of standard-dose liposomal amphotericin B (L-AmB) treatment for a 58-year-old Japanese man with New World cutaneous leishmaniasis (NWCL) caused by *Leishmania braziliensis*. The patient developed a cutaneous ulcer after traveling to Venezuela. Initial treatment with L-AmB (3 mg/kg/day) for 6 days was ineffective, leading to ulcer persistence and later expansion despite subsequent treatment with fluconazole. Even after successful treatment with antileishmanial drugs, the recurrence rate has increased in Latin American countries, where one case study on Leishmaniasis recidiva cutis (LRC) [88], a recurrent form of cutaneous leishmaniasis (CL) previously thought to occur only in Old World cases, was reported. The study reports seven instances of LRC in French Guiana among military personnel treated for CL caused by *Leishmania guyanensis*. Although initial treatment with pentamidine injections cured the lesions, the disease recurred within 3-6 months, manifesting as new lesions around healed scars.

Reoccurrence of the infection cannot be predicted even after complete treatment due to ineffective drugs; a few parasites enter a latent stage in spleenocytes or hepatocytes, which may be inactive, but the risk of reoccurrence is high. In a case study [89] which is related to recurrent cutaneous leishmaniasis (RCL) of an 18-year-old male from north-eastern Brazil, who initially developed an ulcer on his thigh, which healed after treatment with N-methylglucamine. However, two years later, verrucous nodules appeared around the healed scar, indicating a recurrence of the disease. A positive culture confirmed the diagnosis of RCL, and the causative species, *Leishmania braziliensis*, was identified as endemic to the region. The patient was treated with a combination of N-methylglucamine and pentoxifylline, resulting in long-term clinical cure at a 3-year follow-up.

Among immunocompetent hosts, relapse events remain uncommon; however, they are significantly more frequent in immunocompromised individuals, particularly those co-infected with HIV or harbouring *Leishmania RNA virus* (LRV). Co-infection with HIV leads to increased therapeutic difficulty and a higher risk of relapse. A documented case [90] was the first reported in Brazil of a co-infection involving *Leishmania amazonensis*, the causative agent of diffuse cutaneous leishmaniasis (DCL), and HIV. The patient, a 46-year-old man from Maranhão, Brazil, initially presented with a non-healing ulcerative lesion and later developed disseminated erythematous nodules across his body. Diagnosis confirmed *L. amazonensis* as the pathogen, and he was subsequently diagnosed with HIV, with a low CD4+ count indicating a compromised immune system. Treatment included antiretrovirals alongside *leishmaniasis* therapy with liposomal amphotericin B (AmB-L) and Glucantime®, but the patient experienced multiple relapses. In another case, the report describes a 32-year-old Brazilian man with cutaneous leishmaniasis (CL) caused by *Leishmania (Viannia) naiffi*, acquired during military training in the Amazon [91]. The patient’s infection was unusual, as it did not respond to standard pentamidine treatment, which typically succeeds in cases of *L. naiffi*. The lesion was surgically removed to resolve, and subsequent testing identified *Leishmania* RNA virus (LRV) in the isolated parasites. The presence of LRV, which is known to increase parasite virulence in other *Leishmania* species, might explain the patient’s resistance to therapy. A 3-year-old with relapsing visceral leishmaniasis achieved sustained remission after liposomal amphotericin B, pentamidine, and N-methylglucamine combination therapy, supporting multidrug regimens for relapse cases patients [92].

Multiple cases of treatment failure among children under five have also been reported in Colombia [93]. Two such reported cases of visceral leishmaniasis (VL) in Colombia showed resistance to the standard treatment with meglumine antimoniate (Glucantime®) [94]. Usually, standard first-line treatment involves pentavalent antimonial compounds, but both of these children have shown no response to Glucantime® or pentamidine. The report emphasizes the need for alternative treatments due to rising resistance and suggests further research into effective, accessible therapies for endemic regions [95]. Multiple drug treatments have also failed among the patients in Brazil [96], where two cases of recurrent kala-azar (visceral leishmaniasis) by *Leishmania infantum* were reported. Despite attempts with standard treatments (meglumine antimoniate, pentavalent antimonials, liposomal amphotericin B, and pentamidine), both patients experienced recurring symptoms due to drug-resistant kala-azar and severe splenomegaly with hypersplenism, causing frequent hospitalizations [97].

### Europe and Africa

In Europe, only southern countries are affected, due to warm climates that favour sandflies, the high prevalence of infected dogs as reservoirs, and abundant vector species. Limited spread in Northern Europe is due to colder temperatures, lower sandfly survival, and fewer reservoirs. Travel and socioeconomic factors also influence disease transmission, but sustain endemicity mainly in the south. With a high prevalence of asymptomatic human carriers of *L. infantum*, indicating that this parasite poses a latent public health risk [98]. Multiple cases of treatment failure among immunosuppressed patients have been reported in European countries. A 52-year-old immunosuppressed Belgian woman [99] with severe rheumatoid arthritis was diagnosed with visceral leishmaniasis after a bone marrow biopsy revealed *Leishman-Donovan* (LD) bodies. Initial treatment with liposomal amphotericin B succeeded, but she later developed cutaneous leishmaniasis, which was managed with amphotericin B lipid complex. She subsequently experienced relapsing cutaneous lesions and rapidly enlarging lymph nodes, confirmed as *leishmaniasis*. Multiple treatments, including miltefosine, amphotericin B, and N-methyl-glucamine antimoniate, were attempted. Later, a recurrent bone marrow infection was treated with paromomycin and miltefosine. Two years later, she died from leukaemia with severe nasal mucosal destruction. *Leishmania infantum* was identified as the causative agent by PCR. As discussed, some parasites enter a latent stage in splenocytes or hepatocytes. The parasites may be inactive, but the risk of recurrence is high. Similarly, in a case study of a 64-year-old [100] who was infected with cutaneous leishmaniasis 22 years ago, treated partially with antimony. The lesions became disfiguring over time, worsening in summer, leading her to seek medical help. Biopsy showed tuberculoid granulomas and a few *Leishmania* organisms, identified as *Leishmania tropica* by PCR. She was diagnosed with *Leishmaniasis recidiva cutis* (LRC).

A therapeutic failure due to mixed infection with two different strains of *leishmaniasis* has also been a concern, A study has been reported [101], where therapeutic failure in a visceral leishmaniasis patient without HIV was linked to a mixed infection with two different *Leishmania infantum* zymodemes: the rare MON-98, reported for the first time in Greece, and the more common MON-1 from the Mediterranean region. The strains were isolated from two bone marrow samples taken before and 20 days after treatment, as the patient showed no clinical improvement. The MON-98 and MON-1 strains displayed different behaviours and sensitivities to meglumine antimoniate, both *in vitro* and *in vivo*. Mixed infections with distinct *Leishmania* strains could account for variations in disease progression and potential treatment failures in some patients. In 2001, a 4-year-old girl from an area endemic to *Leishmania infantum* in France developed juvenile idiopathic arthritis (JIA) and uveitis. She received immunosuppressive treatments, including prednisolone and anti-TNFα therapies (etanercept, then infliximab). In 2005, after several infliximab doses, she developed visceral leishmaniasis (VL) confirmed by bone marrow examination and PCR. Treatment with liposomal amphotericin B resolved VL, but prophylactic therapy was discontinued due to side effects. In 2007, she developed a nasal granuloma containing *L. infantum* parasites, confirmed by biopsy and PCR, but no systemic relapse occurred [102]. In another case study, an 87-year-old woman with a persistent erythematous forehead lesion was diagnosed with leishmaniasis after histopathological confirmation of amastigotes. Initial treatments with rifampicin and topical paromomycin failed, which may be due to resistant parasites or the patient to the drugs later. Intralesional therapy with n-methylglucamine antimoniate over two months cured the patient [103]. In another case study of treatment failure, a 44-year-old man from southern France with a history of Hodgkin's disease and splenectomy developed visceral leishmaniasis (VL). Bone marrow smears revealed *Leishmania amastigotes* and were confirmed by positive cultures and serological tests; the isolates were identified as *Leishmania infantum* MON-1. Despite multiple treatments, including amphotericin B, meglumine antimoniate, and pentamidine, some cultures remained intermittently positive over months. Eventually, blood and bone marrow cultures turned negative, though lymph node cultures remained positive [94]. Similarly, a 33-year-old pregnant woman from central France presented with pancytopenia and cervical lymphadenopathy, which was confirmed by *Leishmania infantum* infection. She was treated with liposomal amphotericin B, which normalized her blood counts but caused transient skin rashes. Post-treatment, residual lymph node lesions contained *Leishmania* amastigotes [96]. Four cases of visceral leishmaniasis, which include one immunocompetent and three immunosuppressed patients, where the disease has become unresponsive or relapsed after standard treatment with liposomal amphotericin B. These cases highlight the challenges of treating visceral leishmaniasis (VL) in immunosuppressed patients [104]. A 58-year-old kidney transplant recipient experienced multiple relapses despite liposomal amphotericin B (L-AmB) and adjunct therapies. A 15-month-old infant and a 37-year-old HIV-positive male also experienced VL relapses but achieved remission through combined therapies, including miltefosine and meglumine antimoniate. Lastly, a 75-year-old HIV-positive patient with persistent VL and splenomegaly achieved resolution after prolonged meglumine antimoniate treatment. These cases underscore the importance of tailored treatments that integrate combination therapies and immunosuppressive management to achieve sustained parasitological cure in complex VL cases.

## Conclusions

Leishmaniasis remains a major parasitic disease worldwide, particularly affecting vulnerable populations in endemic regions. Although drugs like amphotericin B, miltefosine, paromomycin, and pentavalent antimonials have greatly enhanced treatment efficacy, the rise of drug resistance presents a significant challenge to global health efforts. This review explores the diverse mechanisms underlying drug resistance in *Leishmania* species, including alterations in drug transport, changes in efflux pump activity, genetic mutations, and metabolic adaptations. These resistance mechanisms collectively reduce the effectiveness of current treatments, complicating the management and control of the disease.

Through case studies of treatment failures, it becomes evident that drug resistance in leishmaniasis is not only a biological phenomenon but also a consequence of systemic issues such as inappropriate drug use, inconsistent treatment regimens, and socio-economic factors in endemic areas. For example, cases of relapses and persistent infections despite treatment with liposomal amphotericin B or miltefosine underscore the urgent need for more therapeutic approaches and improved healthcare infrastructure. These treatment failures highlight gaps in our understanding of host-parasite dynamics, immunosuppression, and drug bioavailability, further complicating the clinical management of resistant *Leishmania* strains. The evidence presented in this review underscores the critical need for a multidisciplinary approach to tackle antileishmanial drug resistance. Strengthening pharmacovigilance and treatment monitoring systems is imperative to detect early signs of resistance and guide policy adaptations. Concurrently, research efforts should focus on developing new drugs, repurposing existing drugs, and identifying synergistic drug combinations to overcome resistance mechanisms. Moreover, exploring immunomodulatory therapies and vaccines could provide a complementary strategy to bolster host defences against *Leishmania* infections.

Equally important is addressing the socio-economic factors that perpetuate the spread and severity of leishmaniasis. Enhancing access to healthcare, ensuring the availability of affordable and effective treatments, and implementing community-based awareness campaigns are pivotal to reducing the burden of the disease. Collaborative efforts between governments, global health organizations, and researchers will be critical to achieving these goals.

In conclusion, the fight against leishmaniasis demands a comprehensive, integrated approach that balances scientific innovation with public health initiatives. Understanding the mechanisms of drug resistance and learning from case studies of treatment failures provide valuable insights into the disease's complexities. By leveraging these insights, the global health community can work to develop sustainable solutions that not only curb drug resistance but also improve patient outcomes for those suffering from this neglected tropical disease. While significant challenges remain, the collective efforts of the scientific and medical communities offer hope for a future where leishmaniasis is effectively managed, if not eradicated.
